# Therapy resistance in prostate cancer: mechanism, signaling and reversal strategies

**DOI:** 10.37349/etat.2024.00266

**Published:** 2024-08-29

**Authors:** Neha Thakur, Pallavi Singh, Aditi Bagri, Saumya Srivastava, Vinay Dwivedi, Asha Singh, Sunil Kumar Jaiswal, Sunny Dholpuria

**Affiliations:** University of Bologna, Italy; ^1^Department of Biotechnology, Graphic Era Deemed to be University, Dehradun, Uttarakhand 248002, India; ^2^Amity Institute of Biotechnology, Amity University, Gwalior, Madhya Pradesh 474005, India; ^3^School of Biological and Life Sciences, Galgotias University, Greater Noida, Uttar Pradesh 203201, India; ^4^Department of Life Sciences, J. C. Bose University of Science and Technology, YMCA Faridabad, Faridabad, Haryana 121006, India

**Keywords:** PC, tumor microenvironment, therapy resistance, signaling pathways, molecular mechanisms

## Abstract

Prostate cancer (PC) depicts a major health challenge all over the globe due to its complexities in the treatment and diverse clinical trajectories. Even in the advances in the modern treatment strategies, the spectrum of resistance to the therapies continues to be a significant challenge. This review comprehensively examines the underlying mechanisms of the therapy resistance occurred in PC, focusing on both the tumor microenvironment and the signaling pathways implicated in the resistance. Tumor microenvironment comprises of stromal and epithelial cells, which influences tumor growth, response to therapy and progression. Mechanisms such as microenvironmental epithelial-mesenchymal transition (EMT), anoikis suppression and stimulation of angiogenesis results in therapy resistance. Moreover, dysregulation of signaling pathways including androgen receptor (AR), mammalian target of rapamycin/phosphoinositide 3 kinase/AKT (mTOR/PI3K/AKT), DNA damage repair and Janus kinase/signal transducers and activators of transcription (JAK/STAT) pathways drive therapy resistance by promoting tumor survival and proliferation. Understanding these molecular pathways is important for developing targeted therapeutic interventions which overcomes resistance. In conclusion, a complete grasp of mechanisms and pathways underlying medication resistance in PC is important for the development of individualized treatment plans and enhancements of clinical outcomes. By studying and understanding the complex mechanisms of signaling pathways and microenvironmental factors contributing to therapy resistance, this study focuses and aims to guide the development of innovative therapeutic approaches to effectively overcome the PC progression and improve the survival rate of patients.

## Introduction

Prostate cancer (PC) has become the 3rd most common cause of cancer in men, accounting for nearly 29,430 causalities in the US by the end of 2018 and with an estimation of over 375,000 new cases by the end of 2024 [1–3]. The incidence of PC trended down significantly between 2004 and 2013 by approximately 4.8% and was changed by approx. 8.6% between 2009 and 2013. These averages are usually greater than the trends in all site cancer for males of approx. 1.6% and 2.9% respectively [4]. Patients diagnosed with non-metastatic PC when recorded had a five-year survival rate of nearly 98.9%. However, for those diagnosed with metastatic PC, the five-year survival rate at initial diagnosis was around 28.2% only [4]. The primary instigators of cancer initiation arise from dysregulated molecular signaling pathways and disrupted metabolic functions, which disrupt the regular regulation of cell growth [5]. Despite the utilization of drugs, chemotherapy, and surgical interventions, carcinoma cells have shown remarkable resilience, often re-establishing themselves within the body and manifesting as castration-resistant PC (CRPC) after a certain period of time [6, 7]. Approximately all patients eventually progress to castration resistance, marked by biochemical and radiographic advancements, despite maintaining low levels of blood testosterone due to castration [8]. Patients with PC exhibit a broad range of clinical presentations and outcomes, some patients go into remission, while others advance the illness rapidly to a lethal stage [9].

Since therapies like radiation, chemotherapy, hormonal therapy, and surgery may not be entirely successful in treating advanced or metastatic PC compared to PC stem cells (PCSCs), targeting the androgen signalling system, which is crucial to the development of PC, is a popular use of hormonal treatment. According to study by Attard et al. [10], abiraterone with prednisolone combined with androgen deprivation therapy (ADT) should be considered a new standard treatment for patients with high-risk non-metastatic PC. In metastatic setting, enzalutamide and abiraterone should not be combined for those starting long-term ADT. Clinically important improvements in survival from addition of abiraterone to ADT are maintained for longer than 7 years [10].

However, because undifferentiated PCSCs primarily lack the expression of androgen receptors (ARs), this treatment may not be able to effectively target them [11]. Signalling pathways play a major role in the development and progression of PC. Some signalling pathways like AR signalling pathway are targeted by inhibitors like abiraterone and enzalutamide. Olaparib, an inhibitor of Poly(ADP-ribose) polymerase (PARP). Novel therapies might target changes in epigenetic alterations and alternative splicing, which have an influence on PC development. Comprehending and addressing these pathways is important to create efficacious therapies and enhancing therapeutic results in PC [12]. Patients with PC, they have certain mechanisms of therapy resistance, such as AR overexpression, like tumor cell survival and proliferation [13]. They are caused by high levels of AR in low-androgen conditions during treatment which majorly occurs due to AR gene amplification, epigenetic changes, and miRNA modulation [7].

The tumor microenvironment (TME) in PC is known for its diverse metabolic variations as a result of cancer cell heterogeneity, impacting proliferation and metastasis [14]. In order to sustain their fast growth and survival, PC cells display metabolic modifications inside the TME, such as changes in the metabolism of glucose and amino acids [14, 15]. By providing resources to malignant cancer cells, tumor-associated macrophages, the primary immune cell type in the TME, play a critical role in promoting cancer growth and resistance [14]. In 2018, several new platforms for understanding the complexity and variety of PCs have been made possible by molecular technology and data processing. This will help in the development of methods to counteract invasive and migratory phenotypes beyond AR signalling [1]. In order to treat PC, each category of drug showcase their own mechanism of action, such as ADT, which leads to decrease in male hormone, testosterone, that promotes the growth of PC by inhibiting their action on the AR signalling pathway. Other than ADT, chemotherapy with cabazitaxel and docetaxel, which disrupts cell division and AR signalling by inhibiting microtubule formation, leading to a survival benefit to a survival benefit in advanced PC. Antiandrogens like abiraterone, enzalutamide and apalutamide block the AR or synthesis pathways, preventing testosterone from stimulating cancer growth and increasing survival in advanced PC patients [1]. Reversal strategies are also widely used in PC that aim to overcome treatment resistance and resensitize CRPC cells to antiandrogen therapy. Other prominent reversal strategy is bipolar androgen therapy (BAT), which includes cycling serum testosterone levels from supra physiologic down to near-castrate levels monthly. By re-challenging the tumor cell, BAT has demonstrated the capacity to resensitize patients to earlier innovative AR-targeted medicines, such as abiraterone and enzalutamide [16].

## Impact of the microenvironment on PC

The human prostate gland is a structure, with a central cavity surrounded by neuroendocrine cells, basal cells, and luminal epithelium [17, 18]. Epithelial cells in normal prostate tissues undergo apoptotic cell death by anoikis when they detach from the extracellular matrix (ECM) [19]. Subsequently, the death of these cells is succeeded by the multiplication of naturally occurring precursor epithelial cells [20]. However, in the process of tumor formation and the development of metastatic CRPC, the surrounding tissue may play a role in promoting tumor growth by facilitating the formation of blood vessels, enabling survival mechanisms that resist cell detachment-induced cell death (anoikis), and promoting epithelial-mesenchymal transition (EMT) [21, 22]. Knowing the complex structure of the TME is crucial in unravelling the mechanisms behind therapeutic resistance [23, 24]. By manipulating stromal components, the TME can effectively promote metastases, invasion, and angiogenesis [25]. Within the tumor mass, hypoxia-inducible factor-1 (HIF-1) activates transcription of genes involved in cell survival, glucose metabolism, invasion, and angiogenesis. HIF-1 is composed of an alpha and a beta subunit (HIF-1α and HIF-1β). HIF-1α is hydroxylated by HIF prolyl-hydroxylase, which then targets HIF-1α for degradation under normoxic conditions. Hydroxylated HIF-1α is specifically ubiquitinated by the von Hippel-Lindau E3 ubiquitin ligase, marking HIF-1α for proteasomal degeneration. Under hypoxic circumstances, the hydroxylation of HIF-1α is restricted by the disposal of oxygen molecules and HIF-1α is secured and assembles. HIF-1α can then dichotomize with HIF-β and prompt the transcription of hypoxia-survival genes. Among the transcripts managed by HIF-1 is VEGF [26].

In metastatic disease, notable transformation occurs in the TME as fibroblasts transition into cancer-associated fibroblasts (CAFs) [27, 28]. When CAFs are consistently stimulated under tumor conditions, they secrete alpha-smooth muscle actin (α-SMA), which acts as a biochemical indicator of CAF activity and presence could potentially contribute to the promotion of EMT in PC [29, 30]. Understanding the intricate mechanisms that drive tumor progression and therapeutic resistance is crucial for improving survival rates [21, 31]. By studying the pathways that contribute to the development of the EMT landscape, it can potentially dismantle the microenvironment that supports resistance to treatment in specific tumors [32, 33]. This knowledge may result in small but significant improvements in patient outcomes when combined with existing treatment protocols [34, 35]. The behaviour and biological properties of tumors are shaped by the interplay between the surrounding stromal cells and cancer epithelial cells within the TME [36]. Understanding the intricate workings of EMT is vital in comprehending the intricate mechanisms behind PC’s growth and advancement [37]. EMT enables localized primary tumor cells to develop invasive and migratory abilities, allowing them to spread and establish metastases in distant locations [38]. The fact that androgenic regulation governs this phenotypic process highlights the need to study the TME to understand tumor formation, progression, and ultimately dissemination to other body parts [39, 40].

## Role of common drugs for the treatment of PC

Drug therapy is also known to as chemotherapy which is provided to the patients according to their microenvironment of malignant cells. Some patients require single therapy while some requires combination therapies to fight back the malignancy. Chemotherapy consists of cytotoxic chemicals which suppresses or eradicates the malignant cells in within the body. The primary chemotherapy medication yet utilized is docetaxel, the rest medications are approved after this.

Abiraterone acetate is a chemical compound utilized for the medical treatment to inhibit the activity of cytochrome P450 17α-hydroxylase/17,20-lyase (CYP17A1), an essential enzyme belonging to the cytochrome P450 family [41]. It’s a significant enzyme for the production of androgens. The conversion of pregnanes into steroid hormones follows the biosynthesis of hormones like androgen precursors. It is a hindrance to the progression of PC as it inhibits the synthesis of androgens in glands and also in PC [42]. In 2011, abiraterone acetate was approved by the Food and Drug Administration (FDA) for those patients who are dealing with the advanced PC and who have already gone through the treatment of docetaxel. Further in 2012, it was approved for utilization before therapy of docetaxel [43].

Now the medication named enzalutamide works and competes with the AR. It’s the antagonist which is competitive and is a second-generation medication that got authenticated approval via the FDA after the approval of docetaxel, for the treatment purpose of metastatic CRPC (mCRPC) [44]. Metastasis-free survival is provided via this medication as this enhances the survival rate in patients with non-metastatic CRPC (nmCRPC). The documented side effect of enzalutamide is the compound’s ability to penetrate the brain, such as weariness [45, 46]. Furthermore, the clinical trials are assessed and approved as the enzalutamide is on trial with the combination of different therapeutics like radium-223, docetaxel, abiraterone acetate, etc. [47].

Apalutamide is an antagonist of the AR which is similar to the chemical structure of enzalutamide [48], as it can increase the survival period by 24 months, it has been approved for the treatment of nmCRPC [49].

Here, Table 1 explains the treatment medications used for individuals with PC also the simplified molecular input line entry system (SMILES).

**Table 1:** Medications for treatment of prostate cancer and mode of action

**Sr.No.**	**Drug name**	**SMILES**	**Mode of action**	**Reference**
1	Apalutamide	CNC(=O)C1=CC=C(C=C1F)N1C(=S)N(C(=O)C11CCC1)C1=CC(=C(N=C1)C#N)C(F)(F)F	Antagonism of androgen receptor (AR) signaling by direct binding to the ligand-binding domain, inhibiting DNA binding, AR-mediated gene transcription, and AR translocation from cytoplasm to nucleus, thereby reducing AR availability to interact with androgen response elements (AREs).	[50]
2	Bicalutamide	CC(O)(CS(=O)(=O)C1=CC=C(F)C=C1)C(=O)NC1=CC(=C(C=C1)C#N)C(F)(F)F	Bicalutamide competes with androgen for binding to ARs, thus blocking the stimulatory effect of androgens on prostatic tissue growth.	[51]
3	Cabazitaxel	[H][C@]12[C@H](OC(=O)C3=CC=CC=C3)[C@]3(O)C[C@H](OC(=O)[C@H](O)[C@@H](NC(=O)OC(C)(C)C)C4=CC=CC=C4)C(C)=C([C@@H](OC)C(=O)[C@]1(C)[C@H](C[C@H]1OC[C@@]21OC(C)=O)OC)C3(C)C	Stabilization of microtubules by binding to the N-terminal amino acids of the β-tubulin subunit, promoting microtubule polymerization, inhibiting disassembly, and ultimately blocking mitotic and interphase cellular functions, thereby is preventing tumor proliferation.	[52]
4	Darolutamide	C[C@@H](CN1C=CC(=N1)C1=CC=C(C#N)C(Cl)=C1)NC(=O)C1=NNC(=C1)C(C)O	Competitively inhibits androgen binding to AR, blocking AR nuclear translocation and AR-mediated transcription, resulting in decreased PC cell proliferation and tumor size reduction. It binds more tightly to the AR receptor than other AR antagonists and also acts as a progesterone receptor (PR) antagonist, although the clinical relevance is not fully understood.	[53, 54]
5	Degarelix	CC(C)CC(C(=O)NC(CCCCNC(C)C)C(=O)N1CCCC1C(=O)NC(C)C(=O)N)NC(=O)C(CC2=CC=C(C=C2)NC(=O)N)NC(=O)C(CC3=CC=C(C=C3)NC(=O)C4CC(=O)NC(=O)N4)NC(=O)C(CO)NC(=O)C(CC5=CN=CC=C5)NC(=O)C(CC6=CC=C(C=C6)Cl)NC(=O)C(CC7=CC8=CC=CC=C8C=C7)NC(=O)C	Competitive inhibition of GnRH receptors in the pituitary gland, leading to decreased release of luteinizing hormone (LH) and follicle-stimulating hormone (FSH), which in turn reduces testosterone release from the testes, slowing the growth and reducing the size of PCs.	[55, 56]
6	Docetaxel	[H][C@@]1(C[C@@]2(O)[C@@H](OC(=O)C3=CC=CC=C3)[C@]3([H])[C@@]4(CO[C@@H]4C[C@H](O)[C@@]3(C)C(=O)[C@H](O)C(=C1C)C2(C)C)OC(C)=O)OC(=O)[C@H](O)[C@@H](NC(=O)OC(C)(C)C)C1=CC=CC=C1	Stabilizes microtubules by promoting their assembly and preventing depolymerization, leading to disruption of vital cellular functions, abnormal microtubule formation, and induction of apoptosis by binding to Bcl-2.	[57, 58]
7	Enzalutamide	CNC(=O)C1=C(F)C=C(C=C1)N1C(=S)N(C(=O)C1(C)C)C1=CC=C(C#N)C(=C1)C(F)(F)F	Competitive inhibition of the AR signaling pathway.	[59]

SMILES: simplified molecular input line entry system

In Table 1, which explains the treatment medications used for individuals with PC, SMILES notations were added for several important purpose. SMILES provides a text-based and standardized representation of chemical structures, ensuring stability and exactness in comprising molecular framework of each drug stated, this is mainly helpful for those researchers who wants to briefly study or recreated these structures using computational softwares. In addition, the integration and cross-referencing of data supplied is made easier by SMILES.

## Mechanism of therapy resistance in PC

Chemotherapeutics currently used to treat CRPC include systemic therapies like cabazitaxel and docetaxel, as well as drugs such as abiraterone and enzalutamide that target AR activation either directly or indirectly [31]. Unfortunately, it is not strange for these treatments to encounter primary resistance.

A considerable fraction of patients who are prescribed enzalutamide or abiraterone do not respond well to these drugs [60]. Patients who initially experience benefits from treatment eventually develop drug resistance within 24 months of their first exposure.

### Docetaxel resistance

Understanding the mechanisms behind docetaxel resistance is a frequent challenge caused by the irregular control of molecules involved in cell survival and death [61]. Certain genes, including heat shock protein (*HSP*), signal transducer and activator of transcription 1 (*STAT1*), nuclear factor kappa B (*NF-κB*), *STAT3* and clusterin, have been found to be associated with docetaxel resistance [62, 63]. When GATA-binding factor 2 (GATA2) is upregulated in murine models, it causes an increase in insulin-like growth factor 2 (IGF2) and its downstream targets, which ultimately results in the promotion of docetaxel resistance [64]. On the other hand, decreased p53 activity is associated with a lack of response to docetaxel [65]. The intricate nature of docetaxel resistance is influenced by these mechanisms. Overexpression of certain molecules, including interleukin (IL)-6, IL-8, transforming growth factor-beta 1 (TGF-β1), chemokine (C-C motif) ligand 2 (CCL2), and macrophage inhibitory cytokine 1 (MIC-1), can contribute to the development of docetaxel resistance [21]. These cytokines promote the growth and prevent cell death in PC cells. Interestingly, their release is linked to the effectiveness of docetaxel treatment in patients with CRPC. In addition, there is a correlation between higher levels of β-tubulin isoforms, like taxanes, and the development of resistance to docetaxel [66]. Studies have shown that taxanes have lower binding efficiency to class III β-tubulin isoforms [67]. Additionally, research has found higher expression of class IV β-tubulin and mutations in class 1 β-tubulin, which lead to impaired polymerization in cells resistant to docetaxel [68]. Understanding the mechanisms of multidrug resistance proteins (MDRP), like P-glycoprotein, is crucial in comprehending how these pumps work to expel drugs into the extracellular fluid [69]. This process ultimately diminishes the drugs’ efficacy in targeting cells, resulting in the development of drug resistance [70]. Studies conducted by researchers in the field of neuroscience have demonstrated that the resistance to docetaxel, a chemotherapy drug, can be attributed to the increased expression and phosphorylation of a protein called breast cancer resistance protein (BCRP) [61]. In addition, the sensitivity to docetaxel can be influenced by AR splice variants. Specifically, androgen receptor splice variant 7 (AR-V7) has shown sensitivity to the microtubule stabilization caused by taxanes, whereas tumor xenografts expressing AR-V7 have demonstrated resistance to docetaxel therapy.

### Cabazitaxel resistance

Cabazitaxel, a chemotherapeutic used for CRPC, has garnered significant attention in recent times, however, its efficacy for CRPC is restricted and there are no effective treatments for Cabazitaxel-resistant CRPC [71]. Research has demonstrated that when the ETS-related gene (ERG) is overexpressed in prostate cells, it results in resistance to cabazitaxel both in laboratory settings and in living organisms [72]. Interrupting this interaction can restore sensitivity to cabazitaxel. Research indicates that tumors lacking retinoblastoma exhibit a more favourable response to cabazitaxel treatment in patients with CRPC [73].

### Abiraterone resistance

Re-activation of androgen synthesis in PC cells has been found to be associated with the development of resistance to abiraterone in patients [74]. Understanding the mechanisms behind CRPC progression and abiraterone resistance involves studying the up-regulation and mutations of enzymes in the steroidogenesis pathway. Research has revealed a significant increase in the activity of certain enzymes related to steroid production in lymph node carcinoma of the prostate (LuCaP) cell lines treated with abiraterone. These enzymes include human aldo-keto reductase family 1 member C3 (AKR1C3), CYP17A1, hydroxysteroid 17-beta dehydrogenase 3 (HSD17B3), and SDR5A2 [75]. IL-6 has been found to play a role in the upregulation of steroidogenic enzymes, such as AKR1C3 and HSD3B2. AKR1C3 plays a crucial role in the steroidogenesis pathway and has been proposed as a valuable biomarker for evaluating the advancement of PC [76]. Glucocorticoids are commonly employed to minimize the adverse effects linked to abiraterone treatment. Studies have shown that they can stimulate mutated AR and trigger the growth of PC cells independent of androgens. With abiraterone treatment, the accumulation of androgen precursors has been observed. These precursors bind to mutated AR and trigger downstream signalling [77].

### Enzalutamide resistance

Resistance to enzalutamide in cells is linked to changes in steroidogenesis, glucose metabolism, and autophagy [78]. Enzalutamide-resistant PC cells exhibit an increase in the levels of androgen and its precursors, such as cholesterol, dehydroepiandrosterone (DHEA), and progesterone [79]. Certain genes related to steroid biosynthesis show increased expression, with AKR1C3 playing a significant role. AR mutations in the ligand binding domain have been linked to enzalutamide resistance, affecting around 10–30% of CRPC patients. These mutations lead to an increase in coactivator improvement, changes in ligand specificity and affinity, and the initiation of ligand binding specificity to transition from agonist to antagonist activation [80]. Further investigation is required to determine the clinical significance of the association between the Phe876Leu mutation in the AR and the activation of enzalutamide [81]. Overexpression of p52 leads to enzalutamide resistance, potentially caused by alterations in glucose metabolism and the expression of AR splice variants [82]. Enzalutamide becomes ineffective when there is an overexpression of IL-6, leading to constitutive STAT3 activation.

### Cross resistance

The issue of cross-resistance in PC has emerged as a major concern, greatly reducing the efficacy of treatments in patients who have experienced treatment failure [83]. This resistance applies to all approved treatments for cross-resistance in PC, without any specific class limitations. Research has indicated that individuals who have received prior treatment with abiraterone or docetaxel may experience a decreased response to subsequent enzalutamide treatment, resulting in diminished prostate-specific antigen (PSA) decline and progression-free survival [84]. There may be a lower likelihood of cross-resistance between cabazitaxel and AR-targeted therapies compared to docetaxel, potentially because of variations in how they work [85]. It appears that the effectiveness of docetaxel is reduced when AR-targeted therapies are used, indicating that taxane therapy may have an impact on AR axis modulation. Regardless of the sequence in which docetaxel and AR-targeted therapies are given, cross-resistance is observed [86]. Understanding the development of cross-resistance in PC highlights the importance of utilizing molecules that can effectively target resistance pathways in conjunction with current treatments, ultimately enhancing clinical outcomes [87]. The Table 2 summarizes the key mechanisms of resistance to various treatments used in CRPC aiding in the development of more therapeutic strategies.

**Table 2:** Summarizing the resistance mechanisms

**Mechanism of therapy resistance**	**Description**	**Key findings**	**References**
Docetaxel resistance	Includes irregular control of molecules involved in cell survival and death. Upregulation of GATA2 increases IGF2 resulting in docetaxel resistance. Overexpression of certain cytokines including interleukin (IL)-6, IL-8, TGF-β1, CCL2, and MIC-1 promotes the growth and prevent cell death. AR-V7 has shown sensitivity to the microtubule stabilization caused by taxanes thus contributing resistance.	Genes associated in resistance are: *HSP*, *STAT1*, *NF-κB*, *STAT3* and clusterin. Decreased p53 activity is associated with a lack of response to docetaxel. Higher expression of class IV β-tubulin and mutations in class 1 β-tubulin, has lead to impaired polymerization in cells resistant to docetaxel.	[62–64, 21, 68]
Cabazitaxel resistance	Chemotherapeutic. Overexpression of ETS related gene (ERG) in prostate cells causes resistance to cabazitaxel. Tumors lacking retinoblastoma exhibit a more favourable response to cabazitaxel.	ERG interaction interruption restores sensitivity.	[72, 73]
Abiraterone resistance	Re-activation of androgen synthesis in PC is accompanying with resistance to abiraterone in patients. Steroidogenesis: upregulation of steroidal hormones by IL-6.	Enzymes involved in steroidogenesis are AKR1C3, HSD3B2, and AKR1C3. Glucocorticoids minimizes the adverse effects linked to abiraterone treatment and can stimulate AR and trigger androgen independent cell growth.	[76, 77]
Enzalutamide resistance	Resistance in cells is linked to changes in steroidogenesis, glucose metabolism, and autophagy. Genes related to steroid biosynthesis show increased expression, with AKR1C3. Overexpression of p52 leads to enzalutamide resistance, caused by alterations in glucose metabolism and the expression of AR splice variants.	Exhibits an increase in the levels of androgen and its precursors, such as cholesterol, DHEA, and progesterone. AR mutations in the ligand binding domain affecting around 10–30% of CRPC patients. Overexpression of IL-6 leads to constitutive STAT3 activation, as a result enzalutamide becomes ineffective.	[78, 79, 81, 82]
Cross resistance	Lower likelihood of cross-resistance between cabazitaxel and AR-targeted therapies compared to docetaxel.	Prior treatment with abiraterone or docetaxel may experience a decreased response to subsequent enzalutamide treatment, resulting in diminished PSA decline and progression-free survival.	[83–85]

PSA: prostate-specific antigen; ERG: ETS-related gene; AR-V7: androgen receptor splice variant 7

### Regulation of apoptosis and disease progression in PC

For the inhibition of autophagy and reinstation of docetaxel or ADT response, multiple studies have been done. The fact that autophagy suppression leads to cellular death during anti-cancer therapy supports the encouraging results observed in preclinical animals. Clinical trials have validated the preclinical studies for treatment-resistant cancer [88]. An investigation was conducted by Erkisa et al. [89], for the effectiveness of the combination of palladium, a metal-based chemotherapy medicine, with chloroquine, in which apoptosis is increased in PC cells and the decrement of protein expression is seen with mammalian target of rapamycin/phosphoinositide 3 kinase/AKT (mTOR/PI3K/AKT) [90].

Cancers that depend on the *HIF-1α* gene show that the STK11-AMPK and mitogen-activated protein kinase (MAPK)/p38 pathways work against each other [91]. The MAPK/p38 pathway in PC sustains *HIF-1α* expression. A tissue microarray study is done in PC tissue to evaluate the relationship between STK11 expression and MAPK/p38 activation. In PC, two cell lines were evaluated to understand the significance of the STK11-MAPK/p38 crosstalk. DU145 cells displayed elevated MAPK14 activity throughout the cell proliferation process, whereas PC3 cells showed reduced MAPK14 activity and increased PRKAA1/2 activation. The progressive elevation in phospho-PRKAA1/2 levels in PC3 cells over time was resulted from the administration of the SB202190 inhibitor, which targets the MAPK14-MAPK11 pathway. The activity of STK11 may significantly influence how PC cells react to the inhibition of MAPK14 [92].

## Signaling pathways involved in PC

Despite the current state of treatment, almost all individuals with PC who have metastasized can eventually lead to resistance and disease progression. There are resistance mechanisms that impact segments of the AR axis and additional signalling pathways linked to resistance to therapy. These pathways include the mTOR/PI3K/AKT pathway, the DNA damage repair pathway, and the Janus kinase/signal transducers and activators of transcription (JAK/STAT) pathway.

### AR pathway

**Figure 1:**
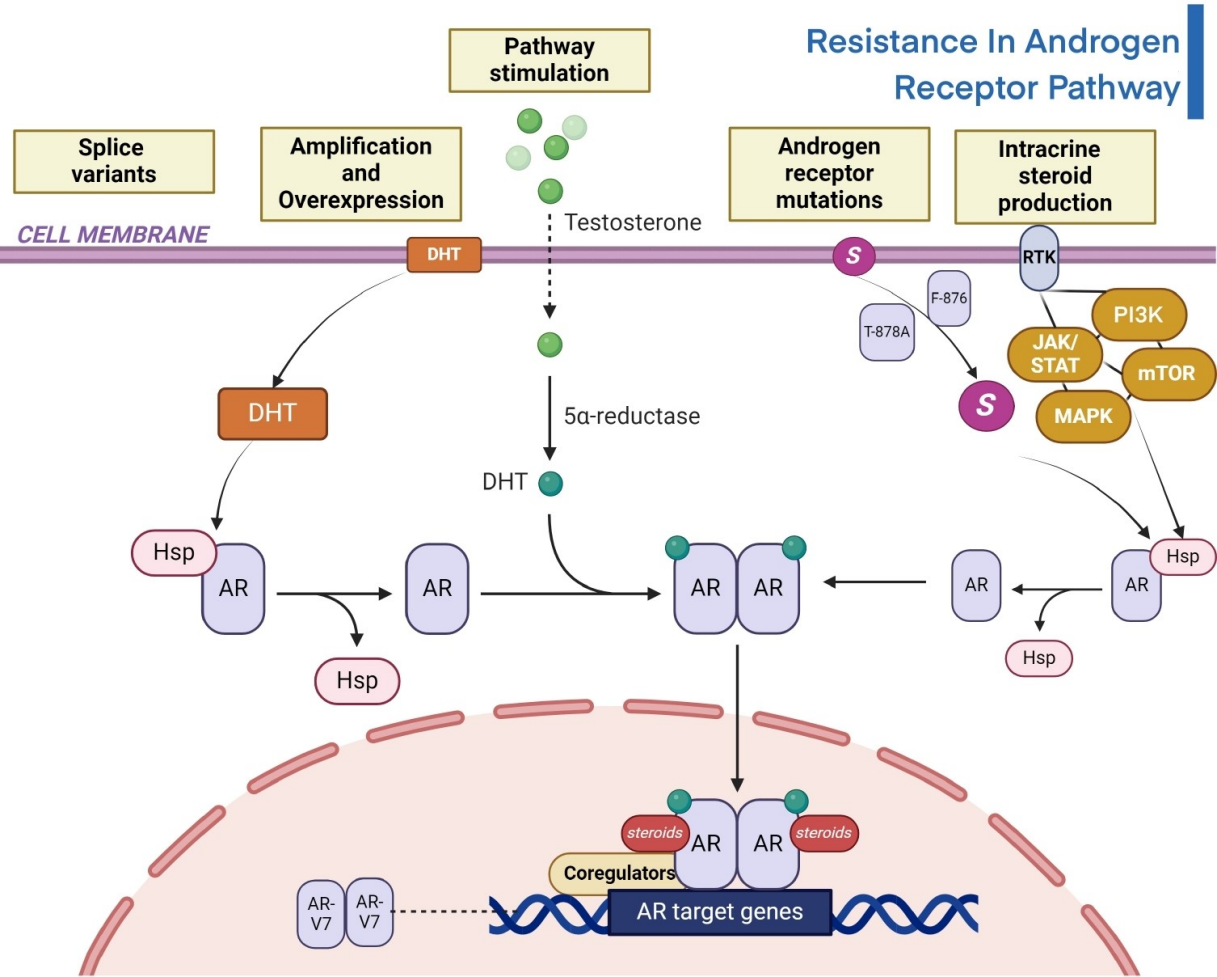
Illustration of resistance in androgen receptor signalling pathway of PC [98, 99]. DHT: dihydrotestosterone; HSP: heat shock protein; AR: androgen receptor; RTK: receptor tyrosine kinase; JAK/STAT: Janus kinase/signal transducers and activators of transcription; PI3K: phosphoinositide 3 kinase; MAPK: mitogen-activated protein kinase; AR-V7: androgen receptor splice variant 7; mTOR: mammalian target of rapamycin *Note.* Adapted from “Androgen Receptor Genomic Pathway”, by BioRender.com. Retrieved from https://www.biorender.com/template/androgen-receptor-genomic-pathway

One of the major clinical concerns regarding metastatic PC is its resistance to therapeutic approaches, which usually results in disease progression even in cases when early treatment success is achieved. This resistance is multifaced, involving diverse mechanisms that impact the AR pathway which is a crucial regulator of the advancement of PC. The steroid hormone containing AR superfamily comprises of three distinct regions. Firstly, it contains the phosphorylation sites that are necessary for its transcriptional activity in the N-terminal domain. Both the ligand-binding domain (LBD) and the DNA-binding domain (DBD) are located in the C-terminal region, which forms the second section [93]. Androgen derivation therapy (ADT), is the main treatment pathway for advanced PC, mainly causes changes in the AR gene, which includes amplification and protein overexpression. Clinical research showcases a considerable increase in AR gene amplification and protein overexpression in ADT-resistant patients compared to those who have not received any treatment have demonstrated that the AR pathway is primarily responsible for developing therapeutic resistance [94]. According to the preclinical models, increased AR expression may make cancer cells more susceptible to supraphysiologic androgen levels, which could result in DNA damage and cytotoxicity. Some patients suffering from PC are resistant to castration (CRPC) demonstrated beneficial responses, like lowered PSA levels, when given a high dosage of testosterone along with conventional therapy [95]. The hypothesis was examined and unexpectedly, individuals treated later with AR-targeted therapies regained their responsiveness, suggesting the potential of this approach in addressing drug resistance. These results emphasize the complex relationship between therapy resistance and the AR-pathway, providing new understanding for therapeutic approaches to combat resistance and enhance patient outcomes in metastatic PC. Various medications such as abiraterone acetate, enzalutamide, apalutamide, and darolutamide encounter resistance through the AR pathway [96, 97]. AR-targeted drugs, that are administered by activating alternative signalling pathways such as the mTOR/PI3K/AKT network, the glucocorticoid receptor route, the serine/threonine-protein kinase B-Raf (BRAF)-MAPK pathway, and the DNA repair pathway, perpetuate this resistance [97]. One of the AR splice variants, AR-V7 is resistant to abiraterone therapy and is not addressed by the current acute respiratory infections (ARIs). The activation of additional signalling pathway, may result in resistance to treatment of abiraterone. Here Figure 1, illustrates the resistance in the androgen pathway.

### mTOR/PI3K/AKT pathway

PC often involves the activation of the mTOR/PI3K/AKT pathway, which facilitates tumor growth, disease progression, and resistance to treatment. PC is significantly linked to genetic changes in phosphoinositide phosphatases, such as phosphatase and TENsin homolog deleted on chromosome 10 (PTEN), which dephosphorylates phosphatidylinositol (3,4,5)-trisphosphate (PIP3) into phosphatidylinositol (4,5)-bisphosphate (PIP2). These changes also control the mTOR/PI3K/AKT pathway. The mTOR/PI3K/AKT pathway influences the development and medication resistance of PC by interacting with important oncogenic signalling cascades such as AR, MAPK and Wingless-related integration site (WNT) [100].

The mTOR/PI3K/AKT pathway when activated can lead to resistance to mTOR/PI3K/AKT pathway directed therapies in PC [101]. In response to Mtorc1 inhibition, certain mechanisms like activation of the rat sarcoma (RAS)/MAPK pathway in used, preventing complete pathway suppression and causing compensatory augmentation of interacting pathways. By inhibiting Mtorc1, we can induce MAPK signalling, that contributes to therapy resistance in PC models. Other than that, feedback loops and redundancy mechanisms within the mTOR/PI3K/AKT pathway can hinder complete pathway inhibition, leading to resistance to targeted therapies [93]. Major contributing variables to PC therapeutic resistance are intrinsic and extrinsic resistance, with activation of oncogenic pathways playing a key role [100].

One factor contributing to PC’s resistance to docetaxel is the PI3K/AKT signaling pathways. Overexpression of epidermal growth factor receptor (EGFR) promotes resistance to therapy in PC. When PI3K/AKT signalling pathway is activated, it leads to resistance to radiotherapy in PC. Overexpression of INPP4B inhibits PI3K/AKT signalling, preventing EMT and reversing docetaxel in PC [100]. Other than docetaxel, abiraterone and enzalutamide can face resistance due to alterations in the PI3K/AKT pathway. In specific patient categories, targeting the mTOR/PI3K/AKT pathway in PC may provide therapeutic advantages, suggesting potential resistance to treatments. Clinical trials with mTOR inhibitors like temsirolimus have shown some clinical benefits in patients with chemotherapy-naive CRPC suggesting a resistance mechanism. Alterations in the AR pathway contribute to resistance to abiraterone and enzalutamide. AR mutations like F876 and T878A can lead to resistance to these drugs. Activation of the PI3K/AKT signalling pathways is associated with resistance to abiraterone and enzalutamide. AR-V7 expression is strongly linked to resistance to hormonal therapies such as abiraterone and enzalutamide. Indeed, AR-V7 could be a useful biomarker in predicting patients, who will benefit from treatment with novel AR blocking agents. Patients with metastatic castrate resistant PC, who are receiving enzalutamide or abiraterone and are positive for AR-V7, have a worse progression free survival and overall survival than those who are negative. However, being positive for AR-V7 is not associated with a significant resistance to taxanes [102]. In PC, mutations in the PI3K pathway have been observed more frequently in metastatic tumors compared to localized tumors, indicating a role in disease progression and resistance. Mutations in the PI3K pathway are more frequently observed in metastatic tumors compared to localized tumors in PC, indicating a role in disease progression and resistance [93].

### DNA damage repair pathway

Homologous recombination repair (HRR) efficiently repairs breaks in the double strands of DNA. HRR pathway in the S and G2 stages of the cell cycle. Other DNA repair mechanisms include base excision repair, nucleotide excision repair (NER), mismatch repair, and non-homologous end joining (NHEJ). NHEJ is faster than homologous recombination and mainly occurs in the G1 phase. Nevertheless, there is recent evidence that NHEJ functions throughout the cell cycle. Beyond the already-known proteins, such as Ku70/80, DNA-PKcs, Artemis, DNA pol λ/μ, DNA ligase IV-XRCC4, and XLF, new proteins are involved in the NHEJ, namely PAXX, MRI/CYREN, TARDBP of TDP-43, IFFO1, ERCC6L2, and RNase H2. Among them, MRI/CYREN has dual role, as it stimulates NHEJ in the G1 phase of the cell cycle, while it inhibits the pathway in the S and G2 phases [103]. PC patients may have mutations in DNA repair enzymes; these mutations range in frequency from 5–10% in localized disease to almost 20% in advanced or metastatic disease [104]. Patients with BRCA2 pathogenic sequence variants have increased levels of serum PSA at diagnosis, an increased proportion of high Gleason tumors, elevated rates of nodal and distant metastases, and high recurrence rates [105]. After being exposed to carcinogens, activation of DNA damage repair mechanisms is essential for avoiding genomic instability and malignant transformation [106]. Biallelic inactivation of CDK12 is associated with a unique genome instability phenotype. The CDK12-specific focal tandem duplications can lead to the differential expression of oncogenic drivers, such as CCND1 and CDK4. As such, there is a possibility of vulnerability to CDK4/6 inhibitors for CDK12-mutated tumors. Moreover, the CDK12 aberrations may be used next to mismatch repair deficiency, as a biomarker of treatment response. This highlights the rationale for the combination therapeutic strategy of immune checkpoint blockade and CDK4/6 inhibition in clinical trials [107]. Platinum-based chemotherapy like docetaxel and cabazitaxel, induces DNA strand breaks, contributing to synthetic lethality in PC treatment [106, 108]. Certain chemotherapeutic medications and radiation can make a tumor more sensitive if DNA repair mechanisms are inhibited. According to study, there is a well-documented overall survival benefit of adding ADT to radiotherapy in localised PC. There is robust evidence indicating that AR activates DNA repair pathways, which provides a rationale behind the use of ADT with stereotactic ablative radiotherapy (SABR) for hormone-sensitive prostate oligometastases [109]. When cancer drugs and DNA repair pathway inhibitors are combined, the susceptibility of tumor cells to these drugs may be increased. DNA repair pathways can contribute to resistance to therapies for PC. The susceptibility of tumors to genotoxic drugs, such as those employed in the treatment of PC, can be increased by inhibiting DNA repair mechanism. The ability of tumor cells to repair DNA can affect resistance to cancer treatments, particularly chemoradiotherapy, which can affect the course of treatment for PC. Certain DNA repair processes, including the NER system, can be inhibited, which can make tumor cells more susceptible to chemotherapy drugs like cisplatin [110]. Tumor cells can develop resistance to cisplatin through higher DNA repair capacity, leading to treatment failure. Inhibition of the NER pathway can enhance tumor cell sensitivity to cisplatin. Individual DNA repair proteins, such as 53BP1, which are involved in NHEJ, affect breast cancer patients’ radio resistance [110, 111]. In individuals with glioblastoma, activation of DNA repair mechanisms, such as ionizing radiation-induced PTEN Y240 phosphorylation, can increase treatment resistance [110].

### JAK/STAT pathway

**Figure 2:**
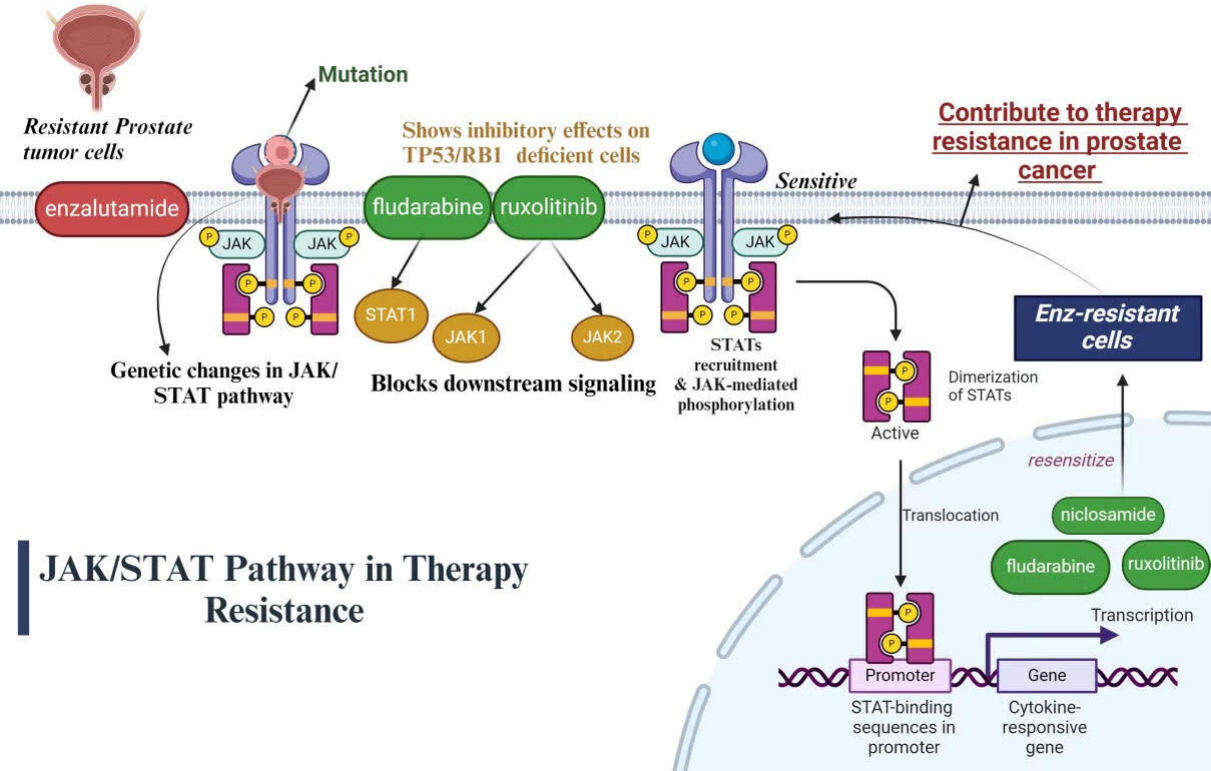
Illustration of JAK/STAT pathway in therapy resistance of PC [115]. JAK/STAT: Janus kinase/signal transducers and activators of transcription; TP53/RB1: tumor suppressor 53/retinoblastoma 1 *Note.* Adapted from “Cytokine Signaling through the JAK-STAT Pathway”, by BioRender.com. Retrieved from https://www.biorender.com/template/cytokine-signaling-through-the-jak-stat-pathway

The treatment and castration-resistant (t-CRPC) phenotype will inevitably arise, even in the cases of initial significant therapeutic success. Data obtained from mice models, clinical specimens and cell models indicates that specific genetic changes, such as deregulation of JAK/STAT system, are connected to t-CRPC. The phenotypes of PC cells resemble those of neuroendocrine glands, which make genetic changes an important factor in the lineage plasticity of cancer [112]. The JAK-STAT signaling pathway is activated in therapy and CRPC, promoting stem cell plasticity and cancer stem cell phenotypes. PC that is resistant to treatment and castration is linked to the features of cancer stem cells and the activation of the JAK1-STAT1-IFIT5 pathway. Certain JAK-STAT small molecule inhibitors, such as fludarabine and ruxolitinib, show remarkable success in treating PC that is resistant to treatment and castration. It can significantly reduce the behaviour of cancer stem cells in vivo and in vitro models by utilising FDA-approved selective small molecule inhibitors to target JAK or STAT1, offering patients additional therapeutic choices [113]. Ruxolitinib, fludarabine, and niclosamide are some of the pharmaceutical inhibitors that target different JAK and STAT proteins. Ruxolitinib blocks downstream signaling by inhibiting JAK1 and JAK2 proteins, which are essential; parts of the JAK-STAT pathway. Ruxolitinib, a JAK1/JAK2 inhibitor, has a greater inhibitory effect on tumor suppressor 53/retinoblastoma 1 (TP53/RB1)-deficient cells. Fludarabine acts as a STAT1 inhibitor, targeting another key protein in the JAK-STAT pathway, to disrupt the signaling cascade [114]. Fludarabine (STAT1 inhibitor) and niclosamide (STAT3 inhibitor) show cooperative roles with JAK1/JAK2 in inhibiting Enz-resistant growth. Enz-resistant cells become sensitive again to JAK-STAT inhibition via filgotinib and ruxolitinib, indicating a universal role in promoting lineage resistance and plasticity through JAK-STAT signaling. In mCRP with TP53/RB1 loss and SOX2 overexpression, JAK-STAT signaling causes lineage plasticity driven by AR-targeted treatment resistance. The overexpression of JAK-STAT signaling genes is facilitated by SOX2’s positive feedback promotion of JAK-STAT signaling. Pharmacological inhibitors such as niclosamide, fludarabine and ruxolitinib that block JAK-STAT signaling have the ability to resensitize Enz-resistant cells, indicating that JAK-STAT signaling may generally contribute to drug therapy resistance in PC The JAK/STAT pathway in therapy resistance is illustrated below in Figure 2.

### Checkpoint inhibitors in MSI-H syndrome

Pembrolizumab checkpoint blockade has been shown to be effective in patients with MSI-H metastatic PC. This is the higher the mutational burden that signifies the tumor is more likely to response with immunotherapy, the MSI-H status [116]. PC was a solid tumor proven to have different MSI-H or dMMR, whereby pembrolizumab, as a PD-1 inhibitor, was approved for use in such solid tumor types. Checkpoint inhibitors resistance to checkpoint inhibitors includes loss of neoantigen expression, mutations in interferon signaling pathways, and induction of alternative immune checkpoints

### PARP inhibitors in patients with DDR pathway mutations

Some PARP inhibitors including olaparib and rucaparib have been approved for patients with mCRPC with alterations in DDR genes such as *BRCA1*, *BRCA2*, and *ATM*. This work is based on the principle of synthetic lethality, which leverages the dependence of cancer cells lacking homologous recombination repair on PARP-mediated repair and are thus susceptible to PARP inhibition. Mechanisms of resistance to PARP inhibitors include secondary mutations that restore homologous recombination, an increase in drug efflux pumps, stabilization of replication forks [117].

Other than the standard drugs like enzalutamide, darolutamide, apalutamide, etc. that focusses on blocking AR signalling in non-metastatic CRPC. Affected individuals receiving these medications as treatment have shown improvement in overall survival [118]. Many ongoing clinical trials like EPI-7386, TRC-253, Compound 28t have focused on positive outcomes by combination therapy or as a standalone clinical trial as well. EPI-7386 is an ongoing treatment option for mCRPC that is undergoing a phase 1 clinical trial to check its efficacy and safety. It can be used as a standalone therapy or in combination with enzalutamide [119].

Clinical trials like BLOERA-2, BELLE-2, PALLAS trial targets the mTOR/PI3K/AKT pathway. A notable trial, BOLERO-2 aims to study the inhibition on pathway along with everolimus and exemestane for postmenopausal women with advanced ER-positive breast cancer. BELLE-2 is another trial that checks the efficacy of PI3K inhibitors—buparlisib with fulvestrant, again in case of postmenopausal women with hormone receptor-positive, HER2 negative advances breast cancer. SOLAR-1 clinical trial investigated the use of alpelisib, a PI3K inhibitor, along with fulvestrant for men and in postmenopausal women with HR-positive, HER2-negative advanced breast cancer [120].

### Changes in the metabolome during PC

In comparison to normal cancer the alterations in the metabolome of PC are characterised crucially. For understanding the progression of disease and diagnosis of potential biomarkers these changes can prove to be significant. Certain metabolites namely sarcosine, show notable elevation during the progression from normal to localized and metastatic PC indicating it as one of the key findings. Sarcosine, an *N*-methyl derivative of glycine is considered as a potential biomarker for aggressive disease and is linked to cancer cell invasion [121]. A distinct metabolic signature in PC has been identified in this research, characterized by elevations in amino acids, lipids, and metabolites associated with stress pathway. Additionally, notable upregulations in the pathways related to amino acid metabolism and androgen-induced protein synthesis has been observed [94]. This suggests that androgens play a significant role in the metabolic reprogramming of PC cells. The study also marks the reduction of metabolites associated with normal prostate function like polyamines and citrate, showing changes in metabolic priorities as cancer progresses.

## Reversal strategies

### Overcoming docetaxel resistance

Various experimental approaches have been investigated to restore the responsiveness of PC to docetaxel [122]. Nevertheless, there is a scarcity of clinical evidence that substantiates these results, or the latter phase studies have shown little effectiveness. One method entails moderating the production of elevated pro-survival and pro-inflammatory chemicals linked to enhanced resistance [123]. Treatment of docetaxel-resistant cell lines with BAY 11-7082, an inhibitor of NF-κB, led to the restoration of sensitivity to docetaxel [124]. Marchantin M, a natural anti-inflammatory chemical derived from liverwort plants, has been discovered to decrease the production of IL-6 and TNFα and deactivate NF-κB. This leads to an enhanced sensitivity of PC cells to docetaxel [125]. Researchers have explored the use of ATP-binding cassette sub-family B member 1 (ABCB1) efflux routes and β-tubulin isoforms as a means to make cells more responsive to docetaxel [123]. Although humans have seven different β-tubulin isotypes, researchers have not fully explained the functional significance of these isotypes [126]. Phase I and II clinical studies have examined the effectiveness of using MDRP-inhibiting medications, including elacridar, in conjunction with chemotherapy [127]. Although phase I studies showed potential, phase II trials only revealed modest therapeutic efficacy.

Utilizing nanoparticles for the transport of docetaxel into cells has been linked to enhanced docetaxel sensitivity. Various categories of nanoparticles have been created, including liposomes, polymeric micelles, and nanoconjugates, with the primary objectives of enhancing selectivity for cancer cells, enhancing drug retention and absorption, and minimizing non-specific toxicity in patients [125]. Studies have shown that docetaxel when attached to magneto-liposomes, may prevent the activation of receptor tyrosine kinase pathways. This indicates that the bound medication has enhanced effectiveness in inhibiting cell proliferation and makes cells more responsive to therapy. Nevertheless, the majority of studies have been carried out in disease models that are not specific to PC, and therefore mostly serve as evidence of the effectiveness of nanoparticles in combating docetaxel resistance in PC.

### Overcoming abiraterone and enzalutamide resistance

Studies indicate that restoring the responsiveness to abiraterone and enzalutamide may be accomplished by modulating disrupted pathways and preventing activation of the AR [128]. Indomethacin, a nonsteroidal anti-inflammatory medication, has been shown to enhance the responsiveness of PC cells that are resistant to enzalutamide. This finding suggests that by targeting intracrine androgens, the effectiveness of enzalutamide treatment may be improved [74]. Furthermore, the use of siRNA with the STAT3 inhibitor AG490 has shown the ability to suppress STAT3 activity, resulting in enhanced apoptosis and inhibition of PC cell proliferation [129]. Targeting the expression of AR variants is a successful strategy for restoring medication sensitivity and reducing the development of CRPC tumors. Niclosamide, an anti-helminthic medicine licensed by the FDA, has been shown to suppress AR-V7 by many mechanisms [130]. These include enhancing the degradation of AR-V7 protein and decreasing the recruitment of AR-V7 to the promoter regions of target genes [131]. The degradation of the protein AR-V7 is caused by pathways that rely on the proteasome [132]. In recent research conducted by Nadiminty et al. [121], it was shown that the downregulation of AR-V7 is associated with the resensitization of enzalutamide. This downregulation occurs via the downregulation of the splicing factor hnRNPA1, which in turn makes resistant cells more responsive to enzalutamide therapy. ASC-J9, a different medication, was discovered to break down both the complete form of the AR and a variant called AR-V3 [133]. This breakdown was linked to a reduction in the development of CWR22Rv1 xenograft tumors. Observations have shown that drugs like EPI and its variants, which target the N-terminal region of the AR, may effectively hinder the proliferation of PC cells. Tumor development in PC xenograft models was decreased by administering EPI in vivo. Niphatenones are a distinct category of medications that only focus on the N-terminal domain of the AR [83]. However, they may not be as effective as other options for treating PC. Furthermore, drug-seq technology is being used to develop and explore new compounds, in addition to treatments that seek to enhance the effectiveness of current drugs. SD-70, a synthetic compound, has been discovered to hinder the movement of PC cells and has shown harmful effects on hormone-sensitive LNCaP cells, C42B cells, and drug-resistant C42B cells in laboratory tests [134]. Additionally, it has shown effectiveness in inhibiting tumor growth in a mouse model using CWR22Rv1 xenografts.

### Neuroendocrine PC

Neuroendocrine PC (NEPC) is an aggressive variant of PC that is defined by characteristics of neuroendocrine differentiation. NEPC can be either a de novo onset (e.g., treatment-naive NEPC) or is induced by long-term ADT and AR pathway inhibitors. Both forms of NEPC are generally nonresponsive to standard androgen axis-targeted therapies, which presents a substantial clinical management problem [135].

#### Treatment-naive NEPC

NEPC is far less common in treatment-naive patients than is adenocarcinoma of the prostate. ICCC often has a high grade poorly differentiated histology and is associated with a worse prognosis. These tumors frequently demonstrate a neuroendocrine phenotype with positive staining for neuroendocrine markers chromogranin A, synaptophysin, and CD56, and a lack of AR expression, explaining their resistance to AR-targeted therapies [136].

#### Treatment-induced NEPC

Treatment-induced NEPC, also known as castration-resistant neuroendocrine PC (CRPC-NE), emerges as a mechanism of resistance following prolonged ADT or AR-targeted therapies like enzalutamide and abiraterone. This subtype is increasingly recognized due to the widespread use of these therapies. It is characterized by lineage plasticity, where adenocarcinoma cells transdifferentiate into neuroendocrine cells under therapeutic pressure [137]. This process involves complex molecular alterations, including the loss of AR signaling, activation of neuroendocrine differentiation pathways, and genomic alterations such as TP53 and RB1 mutations [138].

#### Mechanisms of resistance

The resistance of NEPC to AR-targeted therapies can be attributed to several factors:

##### Loss of AR signaling

NEPC cells often lack AR expression, rendering AR-targeted therapies ineffective.

##### Genomic alterations

Mutations in key tumor suppressor genes like TP53 and RB1 are common in NEPC and contribute to treatment resistance.

##### Lineage plasticity

The ability of prostate adenocarcinoma cells to transdifferentiate into neuroendocrine cells under therapeutic pressure is a critical resistance mechanism.

##### Alternative signaling pathways

NEPC may activate alternative growth and survival pathways, bypassing the need for AR

##### Implications for treatment

Given the resistance of NEPC to conventional therapies, there is a need for alternative treatment strategies. These may include platinum-based chemotherapy, targeted therapies against specific molecular alterations, and novel agents that disrupt neuroendocrine signaling pathways. Research into understanding the molecular drivers of NEPC and developing effective therapeutic approaches is ongoing and critical for improving outcomes for patients with this aggressive form of PC [139].

## Cross resistance

Cross-resistance, a phenomenon seen in PC, has emerged as a notable concern, impeding the efficacy of therapies in patients who have had treatment failure [140, 141]. This resistance is not restricted to any one category of therapies but includes all authorized treatments for CRPC [66]. Research has shown that individuals who have previously had therapy with abiraterone or docetaxel have a decreased response to future enzalutamide treatment [142]. Additionally, PSA reduction and progression-free survival are lowered. Cabazitaxel exhibits lower cross-resistance with AR targeted therapy compared to docetaxel, maybe attributed to disparities in their respective modes of action [143]. The decrease in the effectiveness of docetaxel that is seen after AR targeted treatments implies that taxane therapy may have a function in modulating the AR axis [144]. Regardless of the sequence in which docetaxel and AR targeted treatments are given, cross-resistance develops. The occurrence of cross-resistance in PC highlights the need for the utilization of compounds that hinder resistance pathways as co-treatments alongside current medicines in order to enhance clinical results [145].

## Discussion

Through the process of profiling patients of PC, therapy resistance is not resolved by appropriate strategies and the development of CRPC arises when tumor cells undergo adaptive changes in response to the absence of androgens in the surrounding microenvironment. The cells experience adaptive modifications that lead to heightened synthesis of androgens outside of the testes and the activation of the AR without the need for ligands. Although androgen synthesis inhibitors such as abiraterone may prevent the creation of androgens outside the gonads, a secondary route for the generation of dihydrotestosterone (DHT) remains present. The AR becomes more responsive to low amounts of androgens when it is overexpressed, and this response may be triggered by weak agonists present in anti-androgens. In addition, AR variations enable the AR axis to exhibit ligand-independent constitutive activity, even when the androgen axis and AR antagonists are present. The AR signaling pathway may collaborate with other oncogenic pathways linked to EMT, resistance to cell death (anoikis), and cell survival, hence facilitating the advancement to metastatic CRPC. Taxane-based treatment may induce apoptosis, limit AR nuclear translocation, and impede AR transcription activity, leading to transient disease regression in individuals with metastatic CRPC. Nevertheless, our understanding of the optimal order and amalgamation of therapies for CRPC is restricted. Conventional approaches of categorizing therapy resistant based on Gleason score, PSA levels, and the existence of metastases lack specificity and are inadequate indicators of disease progression. Validating predictive biomarkers is crucial to tailor therapy resistance and assess the effectiveness of treatment.

## Conclusion

CRPC is a highly intricate disease with a range of treatment options. However, the development of chemo-resistance occurs due to disrupted pathways and the activation of AR. Extensive research is being conducted to uncover these intricate pathways and develop strategies to enhance drug sensitivity against therapy resistance. Developing a deeper understanding of these resistance mechanisms will pave the way for the creation of more effective treatment strategies to combat this resistance, which will work in both ways as for combination therapy and in single-drug chemotherapy.

## Abbreviations

ABCB1: ATP-binding cassette sub-family B member 1
ADT: androgen deprivation therapy
AKR1C3: Human aldo-keto reductase family 1 member C3
ARIs: acute respiratory infections
ARs: androgen receptors
AR-V7: androgen receptor splice variant 7
BCRP: breast cancer resistance protein
BRAF: serine/threonine-protein kinase B-Raf
CAFs: cancer-associated fibroblasts
CCL2: chemokine (C-C motif) ligand 2
CRPC: castration-resistant prostate cancer
CYP17A1: Cytochrome P450 17α-hydroxylase/17,20-lyase
DBD: DNA-binding domain
DHEA: dehydroepiandrosterone
DHT: dihydrotestosterone
ECM: extracellular matrix
EGFR: epidermal growth factor receptor
EMT: epithelial-mesenchymal transition
ERG: ETS-related gene
FDA: Food and Drug Administration
GATA2: GATA-binding factor 2
HIF-1: hypoxia-inducible factor-1
HSD17B3: hydroxysteroid 17-beta dehydrogenase 3
HSP: heat shock protein
IGF2: insulin-like growth factor 2
IL: interleukin
JAK/STAT: Janus kinase/signal transducers and activators of transcription
LBD: ligand-binding domain
LuCaP: lymph node carcinoma of the prostate
MAPK: mitogen-activated protein kinase
mCRPC: metastatic castration-resistant PC
MDRP: multidrug resistance proteins
MIC-1: macrophage inhibitory cytokine 1
NEPC: neuroendocrine prostate cancer
NER: nucleotide excision repair
NER: nucleotide excision repair
NF-κB: nuclear factor kappa B
NHEJ: non-homologous end joining
PARP: Poly(ADP-ribose) polymerase
PC: Prostate cancer
mTOR/PI3K/AKT: mammalian target of/rapamycin phosphoinositide 3 kinase/AKT
PIP2: phosphatidylinositol (4,5)-bisphosphate
PIP3: phosphatidylinositol (3,4,5)-trisphosphate
PSA: prostate-specific antigen
PTEN: phosphatase and TENsin homolog deleted on chromosome 10
RAS: rat sarcoma
SMILES: simplified molecular input line entry system
STAT1: signal transducer and activator of transcription 1
t-CRPC: treatment and castration-resistant
TGF-β1: transforming growth factor-beta 1
TME: tumor microenvironment
TP53/RB1: tumor suppressor 53/retinoblastoma 1
WNT: Wingless-related integration site
α-SMA: alpha-smooth muscle actin

## Declarations

### Availability of data and materials

Figures 1 and 2 were created using templates from BioRender.com.

### Copyright

© The Author(s) 2024.
